# Personal

**Published:** 1910-09

**Authors:** 


### PERSONAL

DR. FRANCIS E. FRONCZAK, Commissioner of Health of Buffalo, who attended the International Congress of School Hygiene at Paris in July, spent the remainder of his vacation pleasantly in Europe. He was accompanied by Mrs. Fronczak, and during their tour they passed a pleasant week the guests of Paderewski at his villa on Lake Geneva, returning to Buffalo about August 25.

DR. ROSWELL PARK, of Buffalo, who spent the late spring and early summer in European travel, has returned restored to health and he resumed his professional practice.

DR. CHARLES CARY, of Buffalo, has been in Europe most of the summer enjoying motor tours in Great Britain, and on the continent. He has returned and taken up his professional work.

DR. A. P. SY, Professor of Chemistry at the University of Buffalo, took a trip up the lakes in August, during which he visited the chemical laboratories of the universities and medical colleges at Detroit, Ann Arbor and Chicago. These visits and inspections were in the interest of the new chemical laboratory addition to the University of Buffalo, now in course of construction.

DR. H. C. ROOTH, of Buffalo, sailed August 20, 1910, aboard the Hesperian from Montreal, to spend several months abroad in study and recreation.

DR. SAMUEL SMITH CREIGHTON, University of Buffalo, 1909, and Dr. George Graham Divins, University of Buffalo, 1907, both of Buffalo, have passed the examination for admission to the army medical corps, as assistant surgeons with the rank of first lieutenant. Before assignment to duty they will attend the Army Medical School at Washington, beginning October 1, 1910.

DR. ALEXANDER HUGH FERGUSON, of Chicago, was elected president of the Chicago Medical Society at its last annual meeting, in a keenly contested election. Dr. Ferguson is fitted in an admirable manner to govern this society which is an immense body of men strong in their profession. He entered upon practice in Buffalo in 1881, but soon returned to Winnipeg where he began his medical studies. After a few years he went to Chicago where he became Professor of Surgery in the Post-Graduate Medical School, and later Professor of Clinical Surgery at the College of Physicians and Surgeons, the Medical Department of Illinois State University. Dr. Ferguson lately has been decorated by the King of Portugal as a commander of the Order of Christ.

DR. AND MRS. JAMES WRIGHT PUTNAM, with their son Jack, of Buffalo, are making a motor tour through Canada and New England. They set out early in August, with the expectation of returning in about six weeks.

DR. CHARLES G. STOCKTON, of Buffalo, accompanied by his daughter Miss Lucy, sailed for Europe early in August, on a recreation tour, to last during the remainder of the season.

DR. G. A. HIMMELSBACH, of Buffalo, has been making a three-weeks trip through Canada, fetching up at the seashore for a final stay of some days.

DR. CHARLES STOVER, of Amsterdam, vice-president elect of the Medical Society of the State of New York, became president of the society upon the death of Dr. Charles Jewett, whose obituary is published elsewhere. Dr. Stover is a prominent physician widely known in the eastern part of the state, and is physician to the City and Saint Mary's Hospitals, in Amsterdam.

DR. GEO. B. STOCKER, of Buffalo, announces a change in his office and residence address from 901 to 965 Genesee Street, from August 1, 1910. Hours—8 to 8.30 a. m., 1 to 3 and 7 p. m. Sundays—1 to 3 p. m. only. Both 'phones.

